# Supplementary material for: Differences in the Abundance of Auxin Homeostasis Proteins Suggest Their Central Roles for In Vitro Tissue Differentiation in Coffea arabica
Source: Plants (Basel). 2021 Nov 27;10(12):2607. doi: 10.3390/plants10122607 (PMC8708889; doi:10.3390/plants10122607)
Supplement: Supplementary file 1 [file plants-10-02607-s001.zip › Table S2.pdf]

Table S2. Several protein families involved in auxin homeostasis in *C. arabica*

Protein GH3 family (Group II)

% identity *% similarity	AtGH3.1	AtGH3.2	AtGH3.3	AtGH3.4	AtGH3.5	AtGH3.6	AtGH3.9	AtGH3.17
CaGH3.17a	47 *66	47 *68	48 *68	45 *63	51 *61	50*68	49 *67	**79 *91**
CaGH3.17b	46 *64	27 *32	22 *47	48 *66	48 *79	48*67	45 *64	**61 *75**

Amidohydrolases ILR1

% identity *% similarity	ILR1	ILL1	ILL2	ILL3	IAR3	ILL5	ILL6
CaILR1-Like-1	**76 *90**	72 *85	69 *84	58 *83	75 *88	51 *70	59 *77
CaILR1-Like-2	51*70	53 *72	**55 *73**	50 *69	50 *71	48 *59	51 *71
CaILR1-Like-4	54 *72	64 *80	66 *80	53 *70	**73 *90**	22 *41	49 *69

Protein ABC family (subfamily ABCB)

% identity *% similarity	ABCB1	ABCB2	ABCB11	ABCB19	ABCB21
ABCB2	51 *69	**78 *90**	45 *65	53 *73	45*66
ABCB4	43 *64	43 *62	**65 *79**	44 *64	45*67
ABCB14	44 *65	45 *64	65 *75	46 *66	**74 *86**

The highest identity value that *C. arabica* proteins share with respect to *A. thaliana* is shown in bold and the highest similarity value is shown with an asterisk. Data were obtained by BLAST analysis. For GH3, the analysis was performed with group II proteins involved in auxin conjugation. For amidohydrolases, an analysis was carried out with the proteins of the seven members of the ILR1 family involved in the hydrolysis of auxin. For the ABCB subfamily, the analysis was carried out against some of the proteins related to auxin transport.
